# Impact of the 2020 COVID-19 Pandemic on Older Adults’ Perceived Sedentary Behavior and Physical Activity

**DOI:** 10.1093/geroni/igaa057.3474

**Published:** 2020-12-16

**Authors:** Mikael Anne Greenwood-Hickman, Jacklyn Dahlquist, Julie Cooper, Dori Rosenberg

**Affiliations:** 1 Kaiser Permanente Washington Health Research Institute, Seattle, Washington, United States; 2 Kaiser Permanente Washington, Seattle, Washington, United States

## Abstract

Starting in March 2019, stay-at-home orders meant to control the spread of the COVID-19 pandemic have limited movement, activities, and services in Washington State. For older adults, who are the most sedentary age group, we hypothesized that these public health measures encouraged increased sedentary behavior (SB) and reduced physical activity (PA) levels. To explore this, we conducted 25 semi-structured interviews with a sub-sample of participants in an ongoing sedentary behavior reduction intervention. Interviews were recorded and transcribed, and an iterative coding process was used to extract key themes related SB changes, PA changes, and other impacts of COVID-19 social distancing measures. Most participants reported an increase in SB due to limitations on leaving their home, canceled activities, increased free time in which to pursue indoor hobbies traditionally done in a seated posture (reading, sewing, tv, etc.), and feelings of depression or lack of motivation. Some participants suggested that these restrictions also led to a decrease in their PA and exercise levels due to cancelled fitness classes, loss of social support around exercise routines, and fear of leaving the house. However, other participants reported that the social distancing measures have allowed them to increase their PA levels, giving them more time to walk outdoors or pursue active outdoor hobbies like tennis, gardening, or home improvement tasks. In conclusion, the COVID-19 pandemic and associated social distancing measures have had varying impacts on participant perceived SB and PA levels. It is unclear how these changes will be maintained when pandemic restrictions are lifted.

